# Maternal education is associated with vaccination status of infants less than 6 months in Eastern Uganda: a cohort study

**DOI:** 10.1186/1471-2431-10-92

**Published:** 2010-12-15

**Authors:** Victoria Nankabirwa, Thorkild Tylleskär, James K Tumwine, Halvor Sommerfelt

**Affiliations:** 1 Department of Paediatrics and Child Health, School of Medicine, College of Health Sciences, Makerere University, Kampala, Uganda; 2 Centre for International Health, University of Bergen, Bergen, Norway; 3 Division of Infectious Disease Control, Norwegian Institute of Public Health, Oslo, Norway

## Abstract

**Background:**

Despite provision of free childhood vaccinations, less than half of all Ugandan infants are fully vaccinated. This study compares women with some secondary schooling to those with only primary schooling with regard to their infants' vaccination status.

**Methods:**

A community-based prospective cohort study conducted between January 2006 and May 2008 in which 696 pregnant women were followed up to 24 weeks post partum. Information was collected on the mothers' education and vaccination status of the infants.

**Results:**

At 24 weeks, the following vaccinations had been received: bacille Calmette-Guérin (BCG): 92%; polio-1: 91%; Diphteria-Pertussis-Tetanus-Hepatitis B-Haemophilus Influenza b (DPT-HB-Hib) 3 and polio-3: 63%. About 51% of the infants were fully vaccinated (i.e., had received all the scheduled vaccinations: BCG, polio 0, polio 1, DPT-HB-Hib1, polio 2, DPT-HB-Hib 2, polio 3 and DPT-HB-Hib 3). Only 46% of the infants whose mothers' had 5-7 years of primary education had been fully vaccinated compared to 65% of the infants whose mothers' had some secondary education. Infants whose mothers had some secondary education were less likely to miss the DPT-HB-Hib-2 vaccine (RR: 0.5, 95% CI: 0.3, 0.8), Polio-2 (RR: 0.4, 95%CI: 0.3, 0.7), polio-3 (RR: 0.5, 95%CI: 0.4, 0.7) and DPT-HB-Hib-3 (RR: 0.5, 95%CI: 0.4, 0.7). Other factors showing some association with a reduced risk of missed vaccinations were delivery at a health facility (RR = 0.8; 95%CI: 0.7, 1.0) and use of a mosquito net (RR: 0.8; 95%CI: 0.7, 1.0).

**Conclusion:**

Infants whose mothers had a secondary education were at least 50% less likely to miss scheduled vaccinations compared to those whose mothers only had primary education. Strategies for childhood vaccinations should specifically target women with low formal education.

## Background

Routine childhood vaccinations against tuberculosis, polio, diphtheria, pertussis, tetanus, measles, hepatitis B and haemophilus influenza B have been shown to be effective in protecting children against these diseases in low and middle income countries (LMIC) [1-3]. These vaccinations are highly cost-effective with respect to life years saved [4,5]. Yet, each year, an estimated thirty-four million children do not get vaccinated [4]. In the early 1980's, UNICEF, headed by James P. Grant, spearheaded a child survival campaign that focused mostly on oral rehydration and vaccination, interventions that were seen as measurable [6]. This was followed by a remarkable global increase in vaccination coverage of diphtheria, pertussis and tetanus, from 25% to 75% in ten years [6]. However, this global success was not shared by all and today, about 1.4 million children still die each year from vaccine-preventable illnesses [7].

In fact, thirty years later, only 36% of all one-year old Ugandan children are fully immunized [8] and vaccine-preventable diseases continue to be a major contributor to under-five mortality and morbidity [9,10]. This is despite the fact that the Ugandan Ministry of Health provides free childhood vaccinations and has conducted several national immunization days (NIDs) [11]. The Uganda national expanded programme on immunization (UNEPI) schedule is BCG and polio at birth; polio+ Diphteria-Pertussis-Tetanus-Hepatitis B-Haemophilus Influenza b (DPT-HB-Hib) at 6, 10 and 14 weeks and measles at 9 months [12,13]. In Uganda, vaccination coverage increased in the late 1980 s and the early 1990 s and then stagnated, and even declined in some areas [14]. Several hypotheses for this stagnation such as maternal education have been posited in Uganda and other countries with comparable coverage [15,16]. Mother's education may increase the likelihood of vaccination through increasing knowledge on vaccination. Studies have shown a positive correlation between mother's education and knowledge of vaccination as well as between knowledge of vaccination and acceptance of vaccination [15].

In fact, maternal education may lead to improvements in utilization of primary health care services such as vaccination programmes and other child survival programmes [15-21]. The 2^nd ^millennium development goal is dedicated to universal primary education [17]. The Ugandan government and several other governments in Sub Saharan Africa and South Asia have embarked on an ambitious project to achieve universal primary education by 2015, with some success [17]. In 2007, the Ugandan government launched the universal secondary education scheme. Still, less than 27% of Ugandan women in the reproductive age group have had some secondary school education[8]. This paper compares women with primary school education with those having some secondary school education with regard to the BCG, polio and DPT-HB-Hib vaccination status of their infants.

## Methods

The study was undertaken during a cluster-randomized intervention trial focussed on improving breastfeeding by individual peer counselling (Clinical trials gov: NCT00397150)[18]. Data collection for this study started in January 2006 and ended in May 2008.

### Study site

The study was conducted in Mbale district, in Eastern Uganda, 300 km North-East of Kampala the capital city. The study area is served by Mbale Hospital, which doubles as the district and regional referral hospital. Most of the people were subsistence farmers. With an estimated population of 720,000 [19], Mbale district comprised of 7 counties; the study was conducted in the two biggest counties, namely Bungokho County (rural) and Mbale Municipality (urban). Twenty four clusters were included in the study, 18 rural and 6 urban. Six clusters in Mbale municipality were selected from all its three municipal divisions. Most of the urban areas were large informal settlements. Eighteen clusters in Bungokho County were chosen from eight of its eleven sub-counties. Clusters were included if they neighboured the main road out from Mbale Municipality or were on the 1^st ^or 2^nd ^branch off the main road, had a population of at least 1,000 inhabitants and represented a social and administrative unit.

### Study subjects

Between January 2006 and May 2008, all pregnant women in the selected clusters, were approached by the study team. They were eligible if they resided and intended to stay in the study area, were seven or more months pregnant, intended to breastfeed their infants and consented to participate in the study. In the trial, only singleton children were followed up. Eight hundred and eighty six pregnant women in the study area were identified and all of them were approached.

Written informed consent was obtained from each study participant. Ethical approval was obtained from the Makerere University Research and Ethics Committee, the Uganda National Council for Science and Technology and from the Regional Committee for Medical and Research Ethics for West Norway (REK VEST, approval number 05/8197).

### Data collection

At recruitment, a pre-tested structured questionnaire in the local language (Lumasaaba) was administered by trained data collectors, fluent in the local language. Information was collected on socio-demographic characteristics, antenatal care attendance, marital status and the main source of income. Data was also collected on the current pregnancy and use of bed nets. The pregnant women were followed up until 24 weeks postpartum. After delivery, the mother-infant pairs were visited four times for data collection, at 3, 6, 12 and 24 weeks postpartum. At each of these visits, information was collected on the vaccination status of the children, health seeking behaviour, and breastfeeding practices. The vaccination status of each child was ascertained through inspection of the child's vaccination card by the data collectors. All births, deaths and details of the delivery were recorded within four weeks of delivery.

### Definitions

We categorized marital status into three categories: 'married', 'cohabiting' and other (single, widowed, divorced and separated). In Uganda, it is now common to find couples living together without being formally married and these were classified as 'co-habiting'. Place of delivery was categorised into two groups: 'home', and 'health facility'. Parity was defined as the number of previous live births excluding the study child. Education was grouped into five categories: 'none' '1-4 years', '5-7 years', '8-11 years' and '12 or more years' Primary education was defined as 5-7 years and secondary education as 8-11 years of schooling.

We created a composite index of wealth (socio-economic status) using multiple correspondence analysis (MCA). Because the MCA technique allows combination and ranking of a large number of variables into smaller and fewer variables without prejudgment, it is considered a more accurate indicator of socioeconomic status (SES) than single items such as occupation or possession of particular items. Also, in comparison to principal component analysis (PCA), the MCA technique is more appropriate for discrete variables. This was important in this study because several relevant variables could only be categorical. Furthermore, unlike PCA, which clusters variables together, MCA clusters the categories within these variables together. We used MCA on possession of a TV, radio, mobile phone, chair, cupboard, refrigerator, type of toilet, type of house walls as well as presence of electricity and water in the home. Scores were obtained and categorized into the poorest 20%, middle 40% and richest 40%. Full vaccination at 6 months was defined as having received all the scheduled vaccinations (BCG, polio 0, polio 1, DPT-HB-Hib1, polio 2, DPT-HB-Hib 2, polio 3 and DPT-HB-Hib 3). No-vaccination was not getting any of the vaccines and partial vaccination was having received at least one but not all of these scheduled vaccines.

### Data analysis

Data was directly entered into handheld computers in the field using EpiHandy software (http://www.openXdata.org, version 165.528-142 RC). We analyzed the data with Stata version 9 (StataCorp LP, TX, U.S.). Frequencies and proportions for maternal age, education, parity, wealth, religion, residence, and marital status were calculated. Continuous variables were categorized to avoid doubtful assumptions about linearity. The outcome variables were bacille Calmette-Guérin (BCG) vaccination, polio 0-3 vaccinations and DPT-HB+Hib1-3 vaccinations. The exposure variable was education level. Crude relative risks (RR) and 95% confidence intervals were estimated for the independent variable. We used multivariable generalized linear model (GLM) regression analysis with a log link to estimate the adjusted RR of the exposure variable on the outcome variables. We controlled for place of delivery and household wealth index in the adjusted analysis. In a secondary analysis using multivariable regression, we estimated the effect of maternal education, use of bed nets, delivery at a health facility, household wealth index, mother's age, parity and residence on full (age-appropriate vaccination at 6 months) versus partial vaccination. In both the main and secondary analyses, only variables that were associated with vaccination status yielding a P-value < 0.25 and household wealth index were included in the initial multivariable model. The importance of each of the variables included in the initial multivariable model was assessed based on the Wald statistic and a comparison of each estimated coefficient with the coefficient from the model with only that variable. Variables that did not significantly contribute to the model based on these criteria were eliminated from the final model [20]. The variable household wealth index was included in the multivariable model because of its scientific relevance. Taking the design effect of the PROMISE-EBF clusters into account had no effect on the RR and a negligible effect on precision.

## Results

Of the 886 women approached to participate in the study, 875 (98.8%) accepted to participate. After delivery, 75 participants were not eligible to participate in the study for the following reasons: delivery of stillbirths (17), twin deliveries (17), maternal deaths (2), cleft lip and cleft palate (1), infant deaths up to 24 weeks of age (27) and other reasons (12). In addition, 104 participants were lost to follow up, mostly because of relocation out of the study area. In total, 179 participants were lost to follow up. Of these, 23% had some secondary education compared to 25% of those that remained in the cohort at the end of the study. In both groups, 44% of the mothers had primary education. Data was analysed for the remaining 696 participants.

A total of 696 mother-infant pairs were followed up until 24 weeks post-partum. The mean age of the mothers was 25 years, (range 14 to 44 years) and they had an average of 7 years of formal education (range 0 to 16 years). Mean parity was 3.5 (range 1 to 14). In this cohort, 570 households (82%) were headed by males. Only 177 (25%) of the women earned money for themselves. Most women (72%) had attended at least one antenatal visit by 7 months of gestation. About one half of the women (55%) had been informed about voluntary HIV counselling and testing services, 271 (39%) had been counselled and 212 (31%) had been tested for HIV. In this study, 350 (50%) infants were girls. The 254 children (37%) that were weighed at birth had an average birth weight of 3.4 kg (SD 0.6, range 2 to 6 kg). At 24 weeks, 284 (41%) of the children had received vitamin A. Bed net use by children was 46% (320/696). The distribution of the mothers according to marital status was as follows: 433(62%) were married, 206 (30%) were cohabiting and 57(8%) were single, widowed, divorced or separated.

### Vaccinations

**Figure 1:**
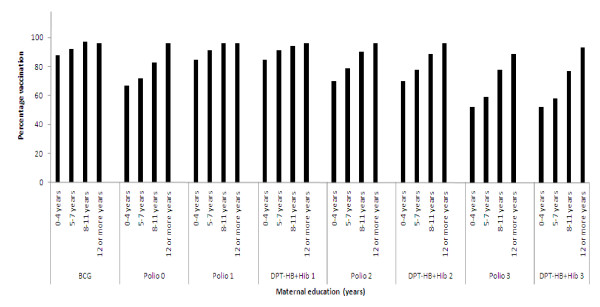
**Maternal education and infant vaccination**.

Out of 696 children, 50 (7%) had not received any vaccination by six months. The remaining 646 children had received some or all vaccinations (Table 1). At 24 weeks, 284 infants (41%) had received vitamin A supplementation. The vaccination coverage increased with increasing level of education (Figure 1).

**Table 1:** Percentage of infants vaccinated at 24 weeks of age in a cohort of 696 infants in Mbale, Eastern Uganda

Vaccine received	N = 696	Proportion vaccinated (95% CI)*
BCG	643	92 (90, 94)
Polio 0	518	74 (71, 78)
Polio 1	630	91 (88, 93)
DPT-HB-Hib 1	627	90 (88, 92)
Polio 2	558	80 (77, 83)
DPT-HB-Hib 2	554	80 (76, 82)
Polio 3	437	63 (59, 66)
DPT-HB-Hib 3	435	63 (59, 66)

* CI indicates confidence interval

### Partial versus full vaccination at 6 months

At 24 weeks, 355 (51%) of the infants had received all the scheduled vaccinations (BCG, Polio 0-3, and DPT-HB-Hib 1-3). Out of the 176 infants whose mothers' had some secondary education, 115 (65%) were fully vaccinated at 24 weeks while 140 (46%) of those whose mothers' only had a primary education were fully vaccinated. The proportion of children with missed vaccinations decreased as maternal education increased. Other factors associated with a reduced risk of missing their vaccinations were delivery at a health facility (RR = 0.8; 95%CI: 0.7, 1.0) and use of a mosquito net (RR = 0.8; 95%CI: 0.7, 1.0). There was no association between vaccination status and wealth index (Table 2)

**Table 2:** Risk factors for partial vaccination at 24 weeks in a cohort of 696 children in Mbale, Eastern Uganda*‡

Characteristic	Number (%)	Unadjusted RR (95% CI) †	Adjusted RR** (95% CI)	Adjusted RRs*** (95% CI)
Mothers education				
None	64 (9%)	1	1	1
1-4 years	124 (18%)	1.0 (0.8,1.2)	0.9 (0.7,1.2)	1.0 (0.7,1.2)
5-7 years	305 (44%)	0.9 (0.7, 1.1)	0.9 (0.7, 1.1)	0.9 (0.7, 1.1)
8-11 years	176 (25%)	0.6 (0.4, 0.8)	0.6 (0.4, 0.8)	0.6 (0.5, 0.8)
12 or more years	27 (4%)	0.2 (0.1, 0.5)	0.2 (0.1, 0.6)	0.2 (0.1, 0.6)
				
Place of delivery				
Home	302 (43%)	1	1	1
Health facility	394 (57%)	0.8 (0.7, 0.9)	0.8 (0.7, 1.0)	0.8 (0.7, 1.0)
				
Use of Mosquito net				
No	376 (54%)	1	1	1
Yes	320 (46%)	0.7 (0.6,0.8)	0.8 (0.7, 0.9)	0.8 (0.7, 1.0)
				
Wealth quintiles				
Poorest 20%	146 (21%)	1	1	
Middle 40%	280 (40%)	1.0 (0.8,1.2)	1.0 (0.8,1.2)	
Richest 40%	270 (30%)	0.9 (0.7, 1.1)	1.1 (1.0, 1.4)	
				
Mother's age				
≤ 19	153 (22%)	1		
20-24	227 (33%)	0.9 (0.7-1.1)		
25-29	165 (24%)	0.9 (0.8, 1.2)		
≥ 30	152 (22%)	1.1 (0.9, 1.3)		
				
Residence				
Rural	554 (80%)	1		
Urban	142 (20%)	0.9 (0.7, 1.0)		
				
Parity				
0	160 (23%)	1		
1--2	210 (30%)	1.0 (0.8, 1.3)		
3--4	163 (23%)	1.3 (1.1, 1.7)		
5 or more	163 (23%)	1.3 (1.1, 1.7)		

† CI indicates confidence interval

‡ Partial vaccination was defined as defined as having received at least one but not all the scheduled vaccinations

* RR < 1 indicate reduced risk of missed vaccination

**Model with maternal education, place of delivery, use of mosquito nets and wealth quintiles

*** Model with maternal education, place of delivery and use of mosquito nets

### Maternal education and vaccinations

**Figure 2:**
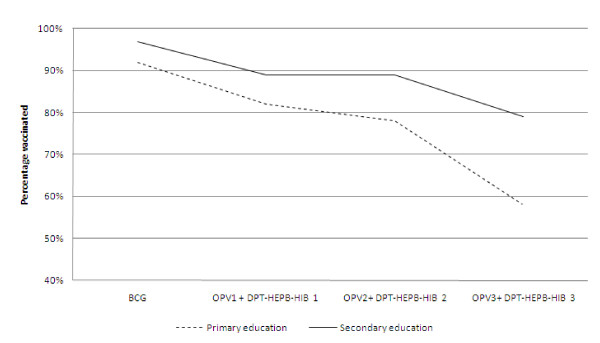
**Primary vs. secondary maternal education and infant vaccination**.

**Figure 3:**
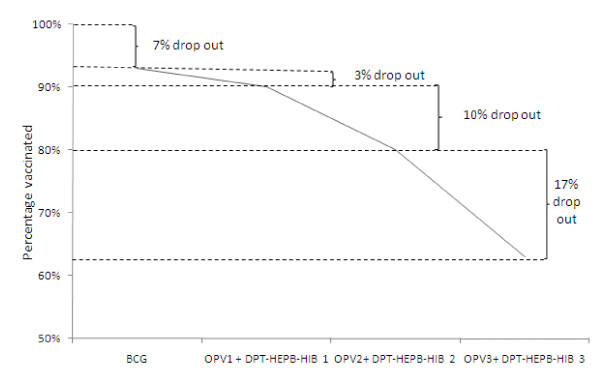
**Percentage drop-out from the immunization schedule**.

Out of the 696 women, 176 (25%) had some secondary education while 305 (44%) had primary education. For all vaccinations (BCG, polio 0-3 and DPT-HB-Hib1-3) women with some secondary education achieved higher vaccination coverage for their infants than women with a primary education (Figure 2). Vaccination coverage dropped steadily from BCG (the first vaccination) to DPT-HB-Hib 3, more so for those with only primary education (Figure 3). At 24 weeks, 177 (58%) of those with primary education had their infants vaccinated with DPT-HB-Hib 3 while 135 (77%) of those with a secondary education had had their children vaccinated with DPT-HB-Hib 3.

Infants whose mothers had a secondary education were less likely to miss polio-0, polio-2, DPT-HB-Hib 2, polio-3, and DPT-HB-Hib 3 vaccines compared to infants whose mothers had received only primary education (Table 3). After adjusting for potential confounders, infants of mothers having some secondary schooling were protected from missing the polio-2 vaccine compared to those whose mothers only had been to primary school (RR: 0.4, CI: 0.3-0.7). In addition infants whose mothers had received some secondary education were 50% less likely to miss DPT-HB-Hib 2 (RR: 0.5, CI: 0.3-0.8), Polio-3 (RR: 0.5, CI: 0.4-0.7), and DPT-HB-Hib 3(RR: 0.5, CI: 0.4-0.7).

**Table 3:** Unadjusted and adjusted risks ratios comparing the vaccination status of 6 month old infants of mothers with only a primary education to infants of mothers with some secondary

Model†	Vaccination	Primary education (n = 304)	Secondary education (n = 176)	Unadjusted RR (95% CI)*	Adjusted RR (95% CI)*
1	BCG				
	No	23 (8%)	6 (3%)	1	1
	Yes	281 (92%)	170 (97%)	0.5 (0.2, 1.1)	0.4 (0.2, 1.0)
					
2	Polio 0				
	No	84 (28%)	30 (17%)	1	1
	Yes	220 (72%)	146 (83%)	0.6 (0.4, 0.9)	0.7 (0.5, 1.0)
					
3	Polio1				
	No	28 (9%)	8 (4%)	1	1
	Yes	276 (91%)	168 (96%)	0.5 (0.2, 1.0)	0.4 (0.2, 0.9)
					
4	DPT-HB-Hib 1				
	No	28 (9%)	10 (6%)	1	1
	Yes	276 (91%)	166 (94%)	0.6 (0.3, 1.2)	0.5 (0.3, 1.1)
					
5	Polio 2				
	No	65 (21%)	18 (10%)	1	1
	Yes	239 (79%)	158 (90%)	0.5 (0.3, 0.8)	0.4 (0.3, 0.7)
					
6	DPT-HB-Hib 2				
	No	66 (22%)	20 (11%)	1	1
	Yes	238 (78%)	156 (89%)	0.5 (0.3, 0.8)	0.5 (0.3, 0.8)
					
7	Polio 3				
	No	126 (41%)	39 (22%)	1	1
	Yes	178 (59%)	137 (78%)	0.5 (0.4, 0.7)	0.5 (0.4, 0.7)
					
8	DPT-HB-Hib 3				
	No	127 (42%)	41 (23%)	1	1
	Yes	177 (58%)	135 (77%)	0.6 (0.4, 0.8)	0.5 (0.4, 0.7)

† Each model is adjusted for wealth index and place of delivery

*CI indicates confidence interval

## Discussion

There were substantial differences in vaccination coverage between women that had a primary education and those with some secondary school education. At 24 weeks, only 46% of the mothers with only a primary school education had their children fully vaccinated compared to 65% of those with secondary school education. Infants whose mothers' had some secondary education were on an average 50% less likely to miss Polio-2, DPT-HB-Hib 2, Polio-3 and DPT-HB-Hib 3 vaccinations. The findings of this cohort study reveal a strong relationship between mothers' education level and the vaccination status of their infants. We also show that this relationship grows stronger as the vaccinations proceed from BCG to DPT-HB-Hib 3. The vaccination coverage is highest with BCG, the first vaccination, and declines steadily with subsequent vaccinations and child age. A similar drop is seen in national estimates [8,21]. In this study, the decline is greatest for those with the least education (0-4 years of education).

Overall, 63% of the children in this study received DPT-HB-Hib 3. This is consistent with the national estimates (63%-68%) in 2007 [22]. However, a coverage of 58% among infants whose mothers only had a primary education is less than the national estimate and also way below both the regional estimate for Sub-Saharan Africa of 73% and the global estimate of 80% [22]. Several studies have shown that maternal education is associated with better utilization of health care services [15,23-26]. Findings from this study are consistent with this. However, we also found that mothers with some secondary schooling made better use of the vaccination programme. Studies elsewhere have also found that levels of knowledge and use of vaccination services are greater for women with at least some secondary schooling [26-29]. The second millennium development goal is dedicated to ensuring that all children everywhere attain a full course of primary education. Indeed targeted investments and political will have resulted in widespread progress, with many regions reaching 90%, and Sub-Saharan Africa reaching 70% school enrolment [17]. However, for optimal vaccination coverage, secondary education may further contribute to enhancing vaccination coverage. Presently, 54% of all children in developing countries attend secondary school while in Sub-Saharan Africa, only a quarter of the children of secondary school age attend secondary school [17]. In order to improve utilization of primary health care services, especially vaccination programmes, our findings indicate that there may be a need to target resources to women with low formal education.

In addition to secondary education of the mother, other factors that were associated with reduced risks of missing scheduled vaccinations were delivery at a hospital or health centre and use of mosquito bed nets. A study in Papua New Guinea reported that 70% of study participants learnt about when to take their children for vaccination from the local maternal and child health (MCH) staff [23]. This could be an important reason for why women who delivered at health facilities were more likely to have their children fully vaccinated.

Wealth was not associated with childhood vaccination in this cohort. Though this finding is similar to other findings in the Philippines [28] and Guinea [30] it contrasts findings in Ghana [31]. Moreover, a systematic review of recent demographic and health survey data from 54 countries found that in comparison to other health care services, vaccination services had the smallest coverage gap between the poorest and richest quintiles of the population [32]. The highly precise estimate of family wealth not being associated with the risk of missing childhood vaccination represents a strong support for The Ugandan Government's policy of providing childhood vaccination services at no direct cost.

In this paper, we did not have data enabling us to adjust for distance to the health centres. Although this is a weakness of the study, it is unlikely to have caused substantial confounding of the results as all the study areas were within 30 minutes by car to a local health centre. Most of these centres vaccinate children on scheduled days. The strength of this study lies in the fact that it was a community-based prospective cohort study with a high follow-up. Only 19% of the mother-infant pairs eligible after delivery were lost to follow-up. There was no significant difference between those lost to follow-up and those that remained in the study with regard to education level. Because it is unlikely that the loss to follow up was unequal by vaccination status, we do not expect it to have substantially biased the estimated risk ratios. Generalizability in this study is limited by the fact that clusters included in this study were close to main roads. We believe these findings can be applicable to regions with similar socio-demographic characteristics because nearly all children in the study villages were recruited into the study. This study was a secondary analysis of data from a cluster randomized trial to improve breastfeeding, and as such, two mothers were not included in the main study because they did not intend to breastfeed. However, it is unlikely that these exclusions would affect our findings. Also, the endpoint of this analysis is 24 weeks of age because that was the endpoint for the breastfeeding trial. To the best of our knowledge, this is one of a few prospective community-based cohort studies on this topic done in a low-income country.

## Conclusion

Women with secondary education were at least 50% less likely to miss their infants' scheduled vaccinations compared to women with only a primary school education. Resources for routine childhood vaccinations should specifically be targeted at women with low formal education.

## Competing interests

The authors declare that they have no competing interests.

## Authors' contributions

All authors participated in the design and plan of the study. Field work was conducted by VN supported by TT and JKT. Analysis and write-up was done by VN, HS, JKT and TT. All authors read and approved the final manuscript.

## Pre-publication history

The pre-publication history for this paper can be accessed here:

http://www.biomedcentral.com/1471-2431/10/92/prepub
